# Successful treatment of gastric cancer after complete response of lung cancer by nivolumab: a case report

**DOI:** 10.1186/s40792-020-01053-y

**Published:** 2020-10-29

**Authors:** Shunji Endo, Yoshinori Fujiwara, Koji Kurose, Masaharu Higashida, Hisako Kubota, Yuko Okamoto, Shumei Mineta, Hironori Tanaka, Toshimasa Okada, Atsushi Tsuruta, Takashi Akiyama, Toru Oga, Tomio Ueno

**Affiliations:** 1 Department of Digestive Surgery, Kawasaki Medical School, 577 Matsushima, Kurashiki, Okayama 701-0192 Japan; 2 Department of Respiratory Medicine, Kawasaki Medical School, 577 Matsushima, Kurashiki, Okayama 701-0192 Japan; 3 Department of Pathology, Kawasaki Medical School, 577 Matsushima, Kurashiki, Okayama 701-0192 Japan

**Keywords:** Stomach neoplasms, Lung neoplasms, Laparoscopic gastrectomy, Nivolumab

## Abstract

**Background:**

Nivolumab is effective for gastric cancer and lung cancer, but complete response is rare. We experienced a case of synchronous gastric cancer and lung cancer who was treated by nivolumab and laparoscopic gastrectomy.

**Case presentation:**

A 63-year-old male consulted our institution and was found to have gastric cancer cT1(SM)N0M0 Stage IA and lung cancer cT2N2M1(PUL) Stage IV. He received eight chemotherapy treatments plus radiation, but the lung disease remained progressive. Finally, he received nivolumab therapy and complete response of both cancers was obtained. The gastric cancer recurred, but was successfully treated by laparoscopic gastrectomy. The resected specimen revealed three lesions, each being pT1aN0M0 Stage IA. The primary gastric cancer seemed to have completely vanished without scarring.

**Conclusions:**

This was thought to be a rare case of gastric cancer recurrence after complete response of gastric cancer and lung cancer to nivolumab.

## Background

Nivolumab is a medication used to treat a number of types of cancer, including melanoma, lung cancer, renal cell carcinoma, Hodgkin lymphoma, head and neck cancer, gastric cancer, and esophageal cancer, among others. Its efficacy for gastric cancer was proven by ATTRACTION-2, a phase III randomized controlled trial (RCT), but the complete response (CR) rate was only 1.1% [1]. Its response for squamous cell non–small-cell lung cancer is also limited, with a CR rate of 1% in the CheckMate 017 phase III RCT [2].

We experienced a rare case with gastric cancer and lung cancer who was treated with nivolumab resulting in CR of both cancers. Gastric cancer recurred, but was successfully treated by laparoscopic gastrectomy.

## Case presentation

**Fig. 1:**
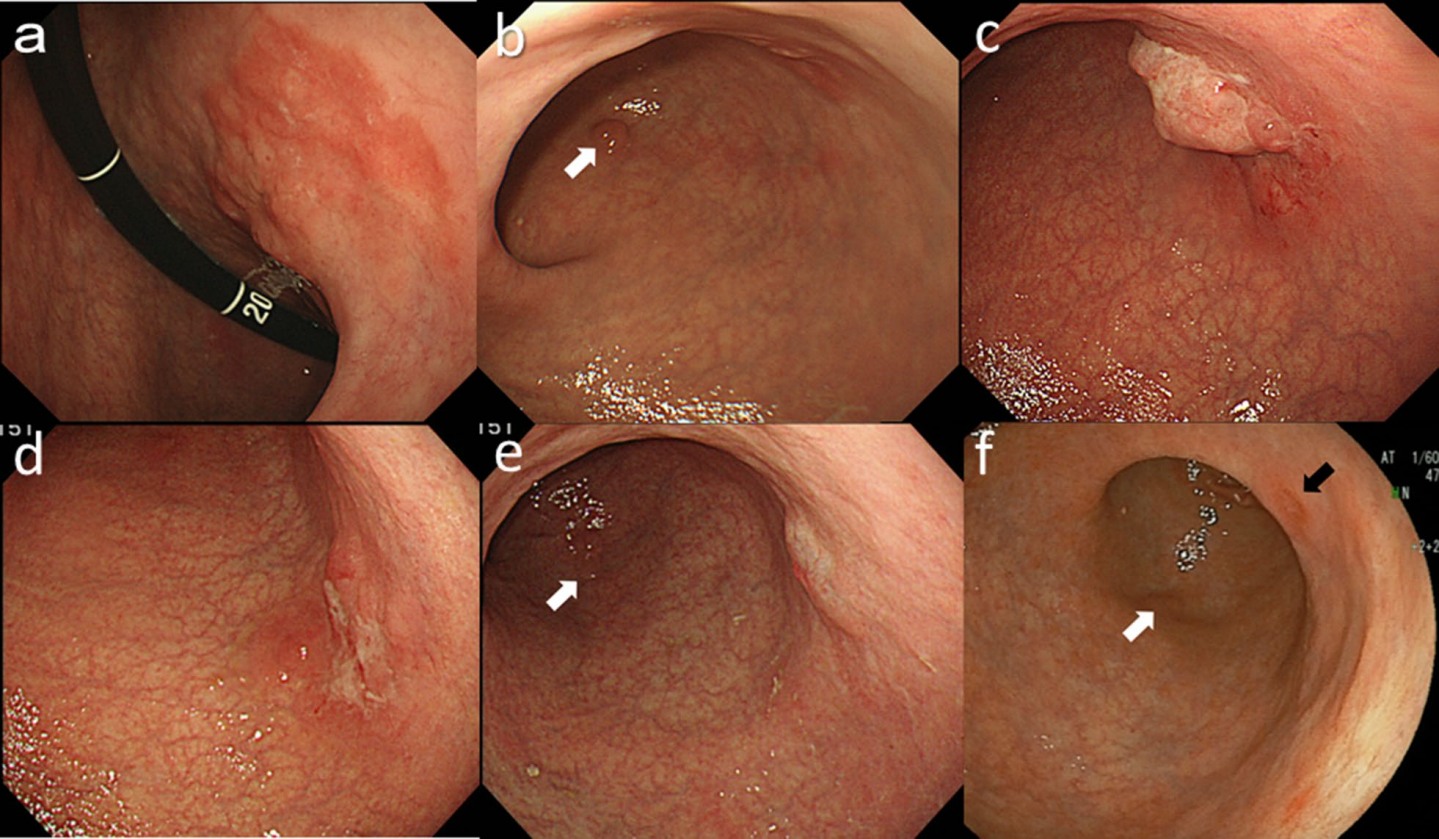
Esophagogastroduodenoscopic findings. **a** Pretreatment: a type 0–IIc tumor in the posterior wall of the gastric angle. **b** Pretreatment: adenoma in the greater curvature of the gastric antrum (white arrowhead). **c** After fifth-line chemotherapy, the tumor grew to type 3 in the posterior wall of the gastric angle. **d** After eighth-line chemotherapy, the type 3 tumor remained in the posterior wall of the gastric angle. **e** After nivolumab, redness and smooth elevation of the posterior wall of the angle were seen and the biopsy revealed no malignancy. Adenoma in the greater curvature of the gastric antrum seemed stable (white arrowhead). **f** Forty-two months after nivolumab, a type 0–IIc tumor appeared in the lesser curvature of the gastric angle (black arrowhead). Biopsy showed adenocarcinoma. Adenoma in the greater curvature of the gastric antrum (white arrowhead) was found to be adenocarcinoma

**Fig. 2:**
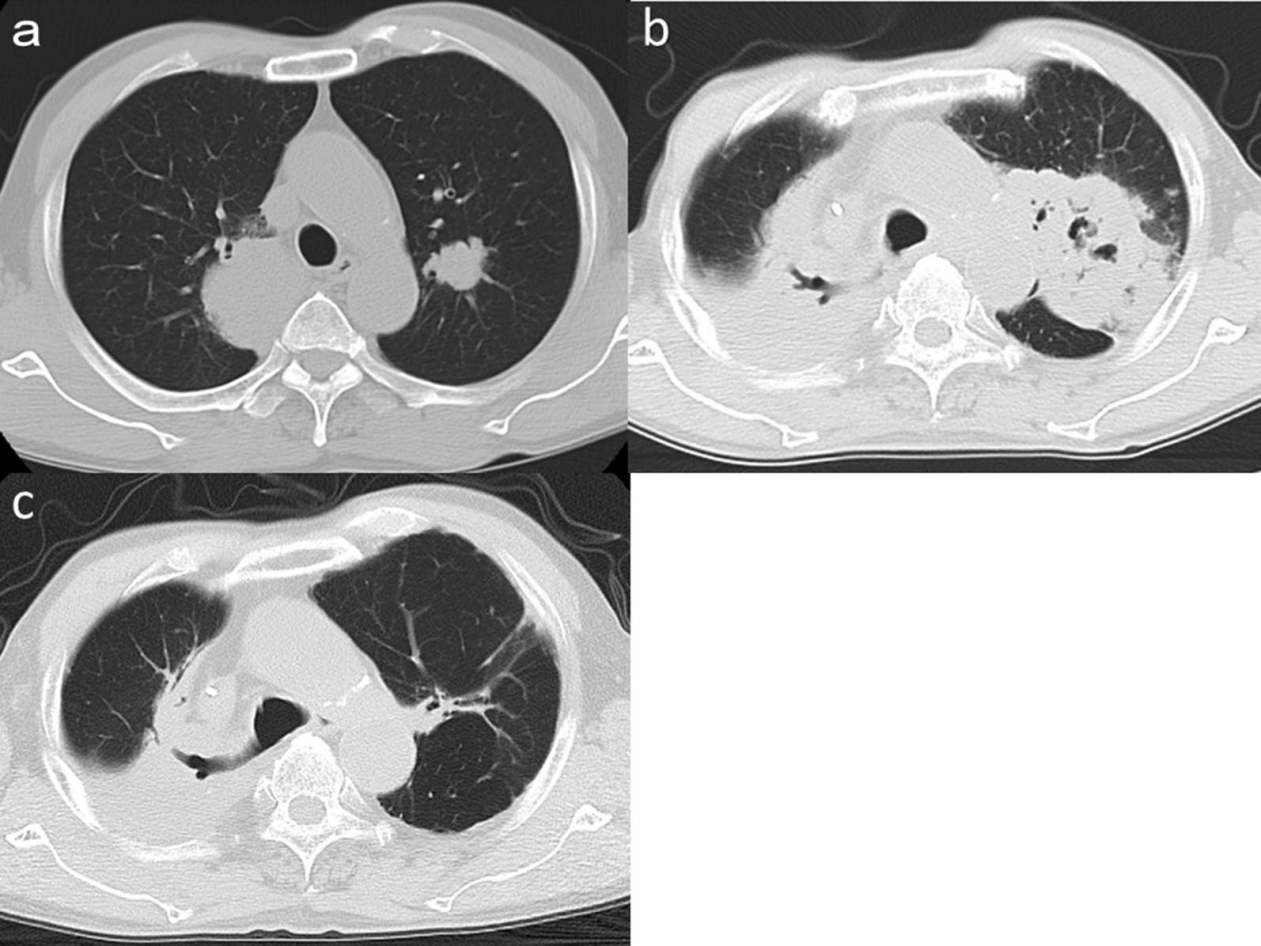
Chest computed tomography findings. **a** Pretreatment, masses of size 5.2 × 4.0 cm in the right upper lung lobe and 2.3 × 2.2 cm in the left upper lung lobe. **b** Before nivolumab, the mass in the left upper lobe grew up to 7.7 × 5.7 cm. **c** After nivolumab, the mass in the left upper lobe disappeared. An atelectasis remained in the right upper lobe

A 63-year-old male visited a nearby hospital with a chief complaint of nausea and epigastric discomfort in March 2009. He consulted our institution in April 2009. Esophagogastroduodenoscopy (EGD) revealed a type 0–IIc moderately differentiated adenocarcinoma in the posterior wall of the gastric angle (Fig. 1a) and a tubular adenoma in the greater curvature of the gastric antrum (Fig. 1b). An abdominal computed tomography (CT) scan showed no lymph node swelling, but a chest CT scan revealed masses of size 5.2 × 4.0 cm in the right upper lung lobe and 2.3 × 2.2 cm in the left upper lung lobe (Fig. 2a). The pretracheal, subcarinal and hilar lymph nodes were swollen. Transbronchial biopsy revealed squamous cell carcinoma. An ^18^F-fluorodeoxyglucose (FDG) positron emission tomography (PET)/CT scan revealed a mass with intense FDG accumulation; the maximal standardized uptake values (SUVmax) were 18.6 in the right upper lung lobe, 22.3 in the left upper lung lobe, and 5.7 in the posterior wall of the gastric angle. The gastric cancer was cT1(SM), cN0, cM0, cStage IA according to the Japanese Classification of Gastric Carcinoma 13th edition [3]. The pulmonary tumors were diagnosed to be right lung cancer with contralateral lung metastasis, staged as cT2, cN2, cM1(PUL), cStage IV according to the General Rule for Clinical and Pathological Record of Lung Cancer 6th edition [4].

**Fig. 3:**
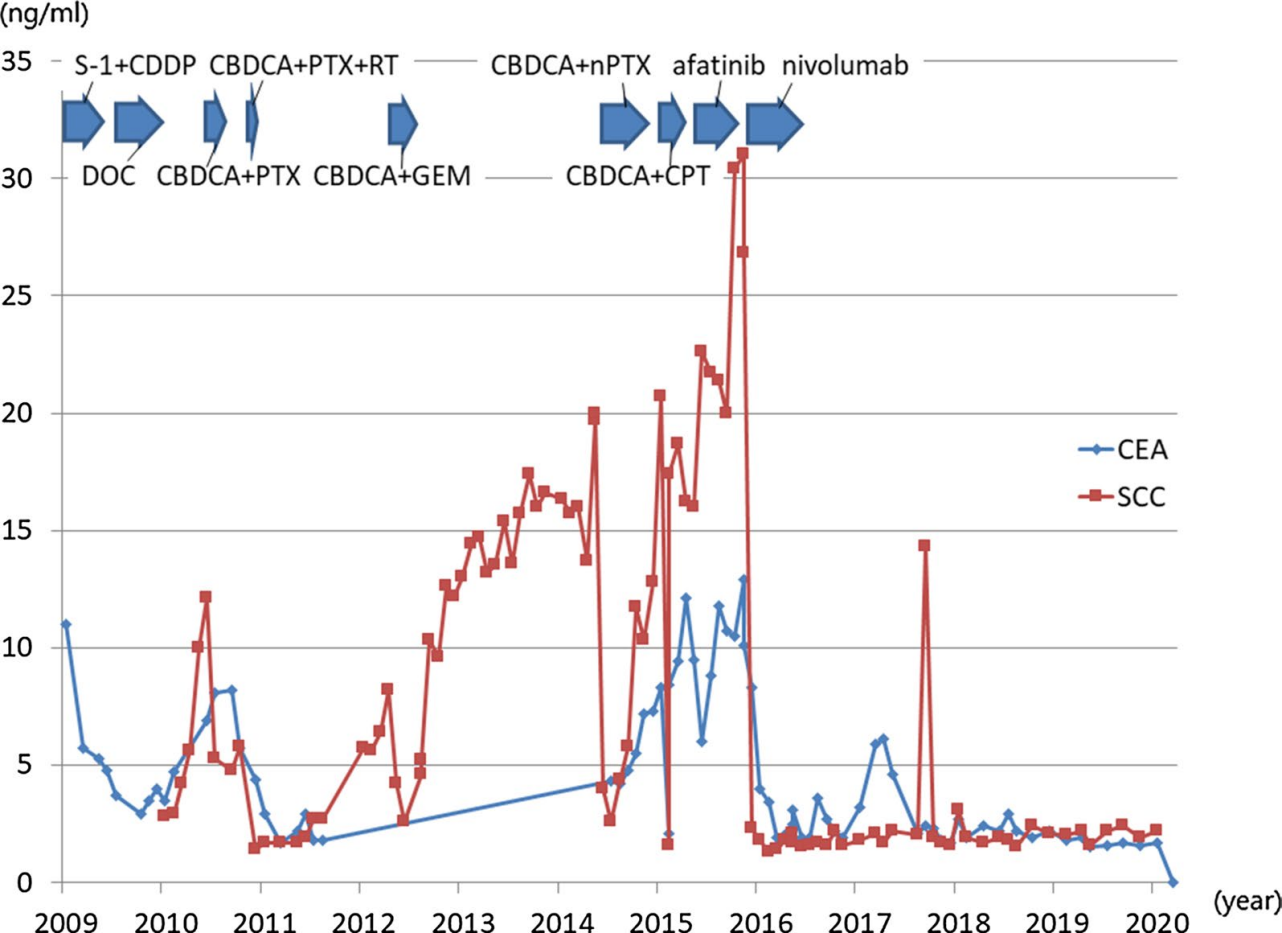
Changes of tumor markers. *CEA* carcinoembryonic antigen, *SCC* squamous cell carcinoma antigen, *CDDP* cisplatin, *DOC* docetaxel, *CBDCA* carboplatin, *PTX* paclitaxel, *RT* radiation therapy, *GEM* gemcitabine, *nPTX* nab-paclitaxel, *CPT* irinotecan

As the lung cancer was advanced and the gastric cancer was early, treatment was focused on the lung cancer. Chemotherapy was administered with four courses of S-1 plus cisplatin, six courses of triweekly docetaxel, three courses of triweekly carboplatin plus paclitaxel, six courses of weekly carboplatin plus paclitaxel with 55 Gy of radiotherapy, and four courses of carboplatin plus gemcitabine. Each chemotherapy regimen was terminated because of progressive disease (PD) of the lung cancer. During this period, EGD was conducted every 3–6 months, and endoscopic stable disease (eSD) was confirmed. During a chemotherapy break between November 2012 and September 2014, PD of the lung cancer and endoscopic PD of the gastric cancer to a type 3 tumor (Fig. 1c) were recognized. Chemotherapy was restarted with six courses of carboplatin plus nab-paclitaxel, then two courses of nab-paclitaxel, four courses of carboplatin plus irinotecan, and 5 months of afatinib. However, these regimens resulted in PD of the lung cancer (Fig. 2b) and eSD of the gastric cancer (Fig. 1d). Finally, nivolumab as the ninth-line setting was administered in February 2016. After ten courses of biweekly nivolumab (3 mg/kg), a chest CT scan showed shrunken lung consolidations of the bilateral upper lobes (Fig. 2c). An FDG PET/CT scan indicated that the lung consolidation of the right upper lobe had an SUVmax of 4.09, which could be well explained by atelectasis. EGD showed redness and smooth elevation of the posterior wall of the gastric angle and biopsy revealed no malignancy (Fig. 1e). From August 2016, he was chemotherapy-free in a good general condition. During this period, chest CT and FDG PET/CT scan showed no progression of the lung cancer. The chemotherapy regimens and the transition of tumor markers including serum carcinoembryonic antigen (CEA) and squamous cell carcinoma antigen (SCC) are summarized in Fig. 3. Transition of cytokeratin 19 fragment (CYFRA) and FDG PET/CT scans are shown elsewhere [5].

However, the type 0–IIa tumor in the greater curvature of the gastric antrum, which had been pathologically diagnosed as a tubular adenoma, was diagnosed as a well-differentiated tubular adenocarcinoma in August 2017, and a type 0–IIc lesion in the lesser curvature of the gastric angle appeared and was diagnosed as a well-differentiated adenocarcinoma in March 2020 (Fig. 1f). It seemed that the 0–IIc tumor had not arisen from the epicenter, but from the margin of the primary gastric cancer. An abdominal CT scan did not show wall thickening of the stomach, swollen lymph nodes, or other distant metastases. An FDG PET/CT scan showed no progression, with an SUVmax of 3.89 in the right upper lung lobe.

**Fig. 4:**
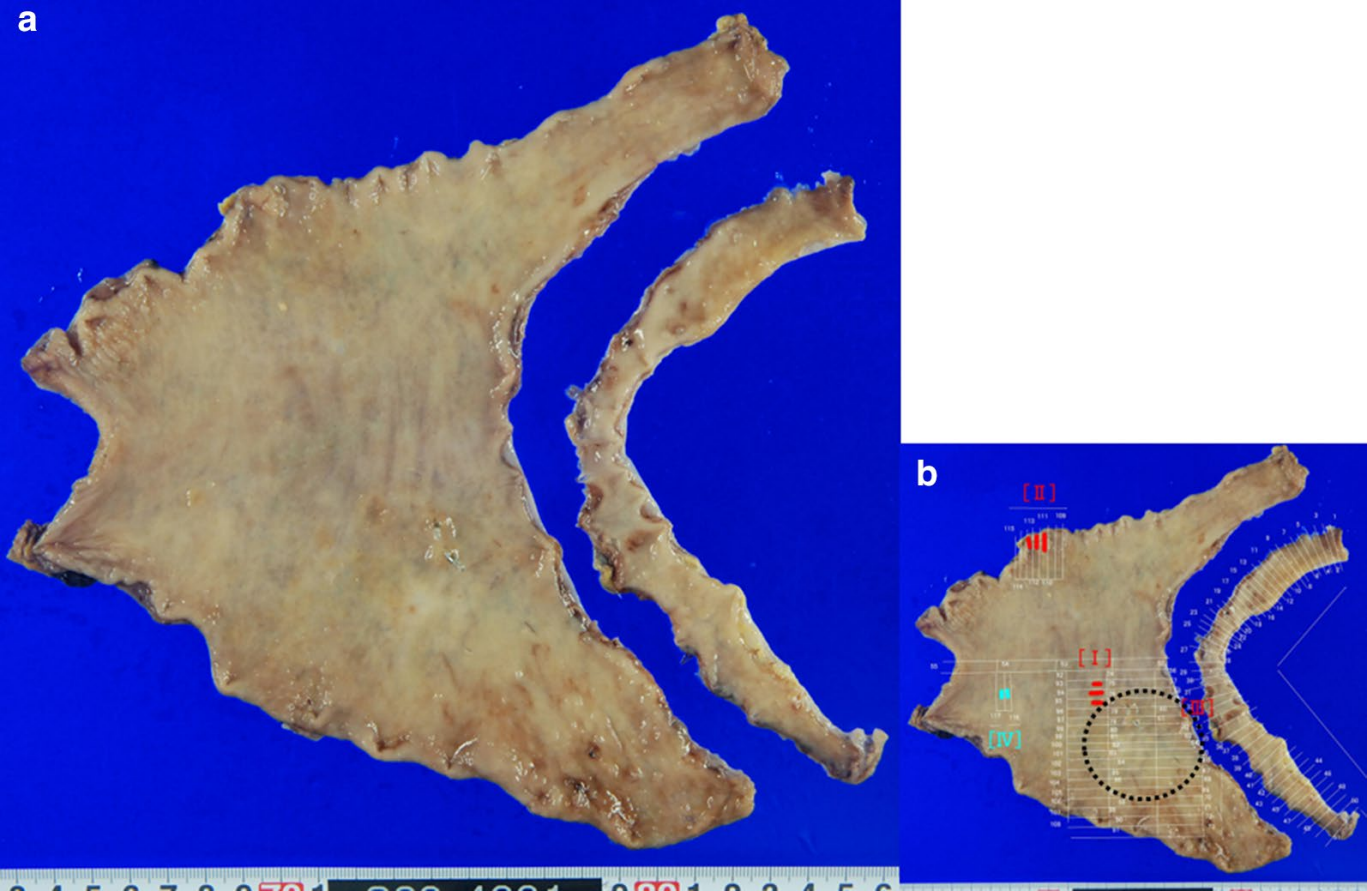
**a** Resected stomach. **b** Mapping of tumors. [I] M, less, Type 0–IIc, 15 × 8 mm; [II] L, Gre, Type 0–IIa, 15 × 12 mm; [III] M, Post, Type 0–IIb, 2 mm (not shown in this figure but in the frozen section); [IV] tubular adenoma, low grade. The black dotted circle indicates the location of the primary gastric cancer, as inferred from pretreatment endoscopic findings

**Fig. 5:**
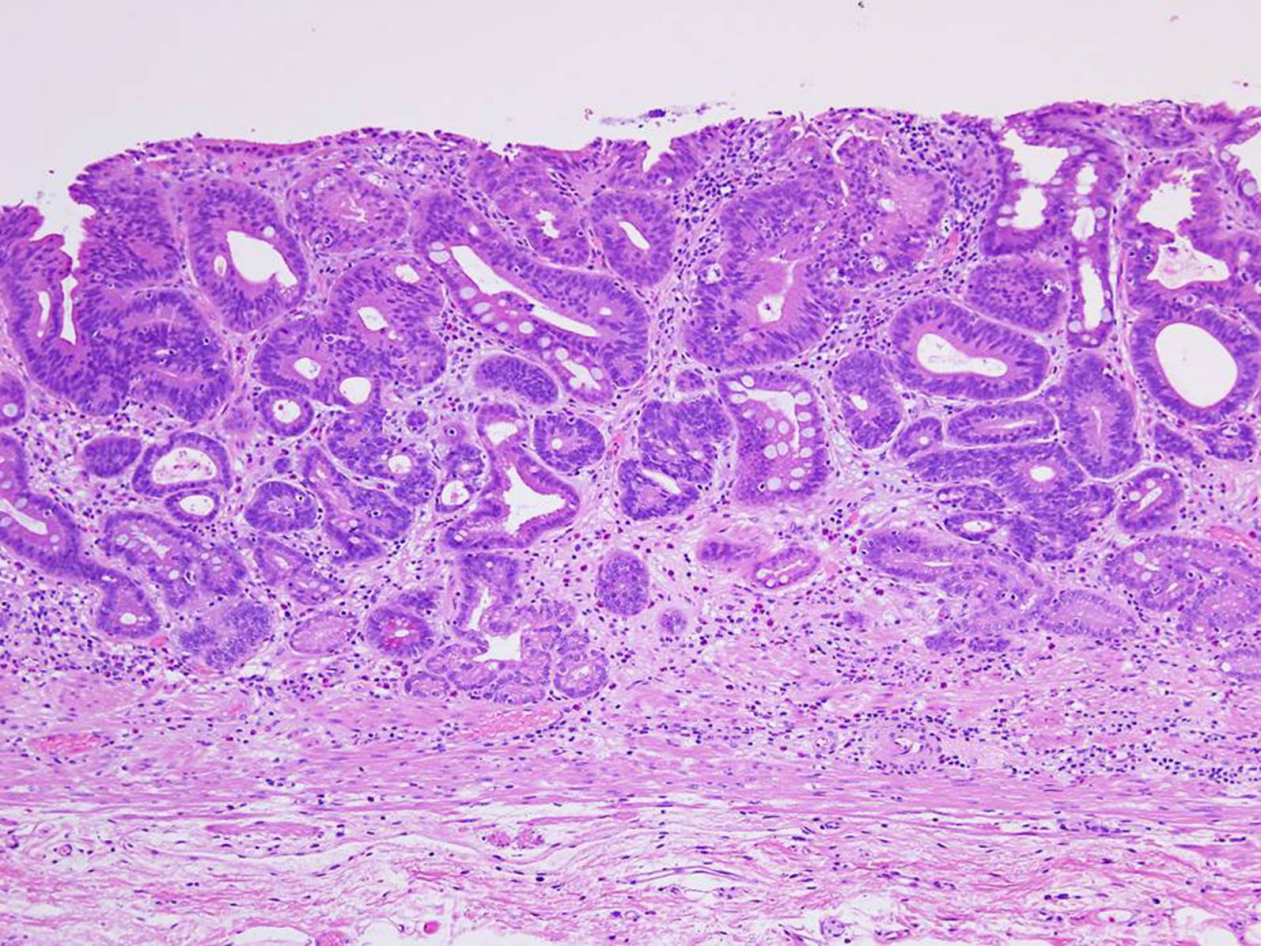
Histopathological findings of the 0–IIc tumor in the lesser curvature of the gastric angle showed well-differentiated adenocarcinoma confined to the mucosa (hematoxylin and eosin staining)

We diagnosed that the lung cancer was in CR and that the gastric cancer had relapsed after endoscopic CR. Preoperative diagnoses were M, less, Type 0–IIc, ycT1a, ycN0, ycM0 ycStage I and L, Gre, Type 0–IIa, ycT1a, ycN0, ycM0, ycStage I according to the Japanese Classification of Gastric Carcinoma 15th edition [6]. Laparoscopic gastrectomy with D1+ lymphadenectomy and Billroth-I reconstruction was performed. As the intraoperative frozen section revealed a small adenocarcinoma on the posterior wall of the proximal resection line, the stomach was additionally resected to confirm a negative proximal margin (Fig. 4a). Histopathological examination revealed three carcinoma lesions and a tubular adenoma (Fig. 4b): [I] M, Less, Type 0–IIc, 15 × 8 mm (Fig. 5); [II] L, Gre, Type 0–IIa, 15 × 12 mm; [III] M, Post, Type 0–IIb, 2 mm; [I–III] tub1, pT1a(M), Ly0, V0, pN0, M0, H0, P0, CY0, pStage IA [6]; [IV] tubular adenoma, low grade. Other than these tumors, no viable tumor cells or signs of tumor regression, including scars, necrosis, fibrosis, granulation, or mucin lakes, were found in the section of the whole posterior wall of the gastric body and the additionally resected stomach. The postoperative course was uneventful. This patient is doing well 1 month after surgery.

## Discussion

The current case obtained CR of gastric cancer and lung cancer by nivolumab. We searched case reports in PubMed using terms of “gastric cancer”, “lung cancer”, and “nivolumab”, with an unlimited publication period, and found one article with synchronous gastric cancer and lung cancer treated by nivolumab. Yamasaki et al. [7] reported a patient with gastric adenocarcinoma (cT1bN0M0 Stage IA) and lung adenocarcinoma (cT1aN3M1a Stage IV) who had achieved a good response for 3 months with nivolumab. In a Japanese article research in Ichushi using the same terms with an unlimited publication period excluding congress abstracts, we found one case report from Nakamura et al. [8] of gastric adenocarcinoma (cT1bN0M0 Stage IA) and lung squamous cell carcinoma (cT4N3M1a Stage IVa), showing that each cancer had ameliorated and that the effect of nivolumab was maintained over 96 weeks. These reported cases and ours involved Stage IA gastric cancer and Stage IV lung cancer. Nivolumab was initially expected to treat lung cancer, but eventually also proved effective for gastric cancer. However, the efficacy of nivolumab for synchronous double cancer is not consistent. Some case reports showed different responses to nivolumab; a case with rapid progression of oral squamous cell carcinoma and PR of lung squamous cell carcinoma [9], and a case with remarkable response of hypopharyngeal squamous cell carcinoma and slight enlargement of lung adenocarcinoma [10].

The resected specimen of the current case showed no remnant of the primary gastric cancer. Cases of gastrectomy after nivolumab treatment are very rare, since nivolumab is indicated for unresectable or recurrent gastric cancer under the Japanese guidelines [11]. PubMed and Ichushi literature searches with the terms “gastric cancer”, “nivolumab”, and “gastrectomy” in an unlimited publication period found one and two reports excluding congress abstracts, respectively. Fujii et al. [12] described the histological feature of the tumor response as massive lymphocyte infiltration. Shiraishi et al. [13] reported a case with a histological response of Grade 1b (slight effect) according to the Japanese classification of gastric carcinoma [6]. As there are no certain specific features of the response to nivolumab according to the literature so far, accumulation of data is awaited.

The current case showed three early gastric cancers in the resected stomach. The type 0–IIa cancer in the greater curvature of the antrum was followed as a tubular adenoma for 11 years without progression. There are two possible explanations for this long-term stability; it was originally adenocarcinoma and remained unchanged by chemotherapy, or it transformed from adenoma during follow-up. The other two lesions seemed to exist around the primary 0–IIc cancer in the posterior wall of the gastric angle (Fig. 4b). They may have arisen from remnant cells of the primary cancer. However, sectioning of a wide area revealed no evidence of the primary cancer or even lymphocyte infiltration. It is uncertain whether nivolumab can cure gastric cancer completely without a trace, but these two lesions might have been heterochronous gastric cancer after CR of the primary gastric cancer, rather than relapses of the primary gastric cancer.

In conclusion, we experienced a patient with synchronous gastric cancer and lung cancer. Nivolumab was effective for both cancers, achieving CR. Although the gastric cancer recurred 3.5 years after nivolumab treatment, it was successfully resected.

## Data Availability

Not applicable.

## Abbreviations

RCT: Randomized controlled trial
CR: Complete response
EGD: Esophagogastroduodenoscopy
CT: Computed tomography
FDG: Fluorodeoxyglucose
PET: Positron emission tomography
SUVmax: Maximal standardized uptake values
PD: Progressive disease
eSD: Endoscopic stable disease
CEA: Carcinoembryonic antigen
SCC: Squamous cell carcinoma
CYFRA: Cytokeratin 19 fragment
